# A Label-Free Fluorescent Amplification Strategy for High-Sensitive Detection of *Pseudomonas aeruginosa* based on Protective-EXPAR (p-EXPAR) and Catalytic Hairpin Assembly

**DOI:** 10.4014/jmb.2405.05006

**Published:** 2024-06-14

**Authors:** Lu Huang, Ye Zhang, Jie Liu, Dalin Zhang, Li Li

**Affiliations:** 1 Interventional Therapy Department, Changsha Fourth Hospital, Changsha, Hunan Province 410006, P.R. China; 2 Cardiovascular Medicine Department, Changsha Fourth Hospital, Changsha, Hunan Province 410006, P.R. China; 3 Nursing Department, Changsha Fourth Hospital, Changsha, Hunan province 410006, P.R. China

**Keywords:** *Pseudomonas aeruginosa* (*P. aeruginosa*), thioflavin T (ThT), G-quadruplex, protective-EXPAR

## Abstract

This study presents a fluorescent mechanism for two-step amplification by combining two widely used techniques, exponential amplification reaction (EXPAR) and catalytic hairpin assembly (CHA). *Pseudomonas aeruginosa* (*P. aeruginosa*) engaged in competition with the complementary DNA in order to attach to the aptamer that had been fixed on the magnetic beads. The unbound complementary strand in the liquid above was utilized as a trigger sequence to initiate the protective-EXPAR (p-EXPAR) process, resulting in the generation of a substantial quantity of short single-stranded DNA (ssDNA). The amplified ssDNA can initiate the second CHA amplification process, resulting in the generation of many double-stranded DNA (dsDNA) products. The CHA reaction was initiated by the target/trigger DNA, resulting in the release of G-quadruplex sequences. These sequences have the ability to bond with the fluorescent amyloid dye thioflavin T (ThT), generating fluorescence signals. The method employed in this study demonstrated a detection limit of 16 CFU/ml and exhibited a strong linear correlation within the concentration range of 50 CFU/ml to 10^5^ CFU/ml. This method of signal amplification has been effectively utilized to create a fluorescent sensing platform without the need for labels, enabling the detection of *P. aeruginosa* with high sensitivity.

## Introduction

Nosocomial infections, one of the most challenging and common infections to treat due to their restricted susceptibility to antimicrobial agents, are predominantly caused by *Pseudomonas aeruginosa* (*P. aeruginosa*) [1, 2]. *P. aeruginosa* is a prevalent pathogen responsible for infections among patients undergoing post-operative care [3, 4]. In severe cases, it can result in systemic infection, disrupt the intended therapeutic effect, and cause delayed postoperative therapy [5-7]. By forming biofilms, it also induces infections in burn incisions and on implanted devices (*e.g.*, urinary catheters). As a result, early and precise detection of *P. aeruginosa* is extremely beneficial for guiding the nursing care strategy of surgical patients and enhancing prognosis.

The current methods used to identify *P. aeruginosa* include traditional culture-based techniques [8, 9], enzyme-linked immunosorbent assay (ELISA) [10, 11], immunofluorescence [12], isothermal amplification strategies [13, 14], and polymerase chain reaction (PCR) [15, 16]. For example, the classic culture-based approach has a long turnaround time of 3-7 days. The antibody-based ELISA method is prone to significant variability between batches and is expensive. The PCR method requires advanced instruments and expert personnel. Hence, in order to address those challenges, there is a pressing requirement for novel, robust instruments that are both swift and highly responsive in detecting *P. aeruginosa*. These tools should also provide guidance to individuals involved in implementing appropriate control measures. The initial identification of an aptamer occurred approximately thirty years ago through the utilization of an in vitro evolutionary technique known as systemic evolution of ligands by exponential enrichment (SELEX) [17-19]. Since then, a multitude of aptamers have been created for the identification of different substances using a range of diagnostic or bio-sensing tests, thanks to their obvious advantages over antibodies, including : (1) the cost of synthesizing of aptamer is minimal, (2) it can be easily modified with different moiety such as fluorophores and nanomaterials, (3) it has a strong affinity to the target,(4) it has a high level of specificity towards the target, and (5) it is stable at room temperature. Over the past few years, numerous detection methods utilizing aptamers have been developed to efficiently and reliably identify *P. aeruginosa* by incorporating fluorescence signals [20, 21]. Nevertheless, the methods described above still have several drawbacks, such as i) inadequate sensitivity and ii) the requirement to label fluorescent dyes, which are prone to experimental environmental interference (*e.g.*, ph and temperature).

The exponential amplification reaction (EXPAR) has garnered significant interest in the field of bio-sensing due to its exceptional efficiency in amplifying signals [22, 23]. EXPAR is initially constructed using a template DNA (T-DNA) that consists of two repeat regions that are divided by a short nicking endonuclease (NEase) recognition sequence. These repeat regions are specifically engineered to match a trigger sequence. Following a single round of circular extension and subsequent cutting facilitated by DNA polymerase and NEase, two replicas of the trigger DNA are generated. As a result, it is capable of achieving exponential signal amplification [24, 25]. However, the accuracy of the EXPAR based approaches remains to be a significant obstacle because of the non-specific binding between interfering sequences and the template.

**Fig. 1:**
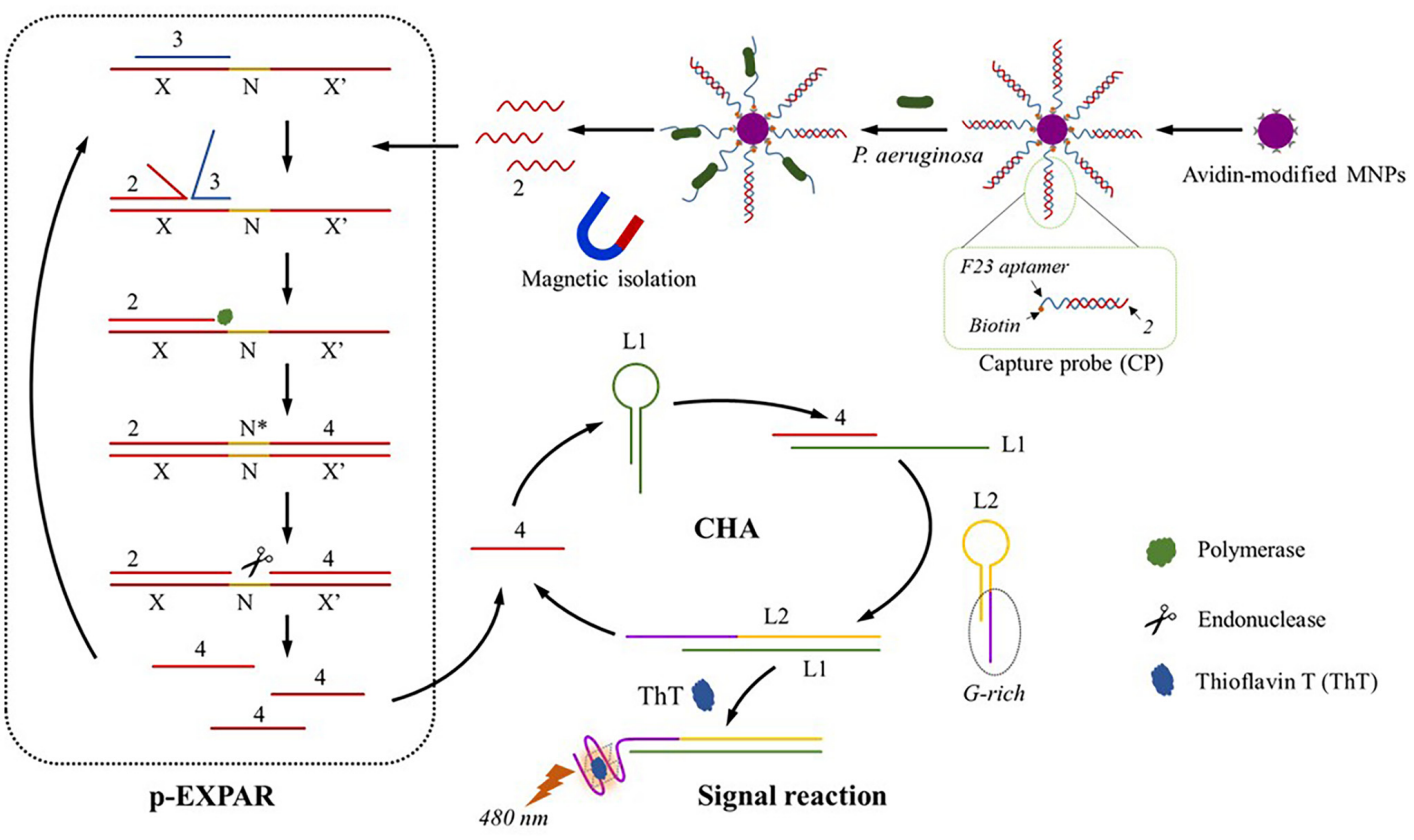
The working principle of the proposed method for *P. aeruginosa* detection based on protective- EXPAR and CHA.

Here, we developed a two-step signal amplification fluorescence biosensor that is both sensitive and label-free for the detection of P using protective-EXPAR (p-EXPAR) and CHA (Fig. 1). The F23 aptamers/”2” duplex was linked to magnetic nanoparticles (MNPs) using the streptavidin-biotin method in this test. Based on the concept of competition, aptamer is desirable to combine both *P. aeruginosa* and the “2” is released from the capture@MNPs. The released “2” can then be collected via magnetic separation. Afterwards, the liquid above the sediment contained only unattached “2” was introduced into the EXPAR reaction system. The “2” has the potential to form a complementary pair with template DNA (T-DNA) and subsequently release the “3” from the template. The “3” was employed to safeguard the T-DNA from hybridizing with non-specific sequences and to enhance the specificity of the EXPAR-based amplification (protective-EXPAR). The “2” was subsequently elongated using DNA polymerase and dNTPs. The nicking enzyme is capable of creating a gap at the recognition site, allowing the DNA polymerase to proceed with extending from the gap and replacing the subsequent DNA strand. This technique was iterated multiple times, resulting in the efficient amplification of the “2” sequence. A significant quantity of single-stranded DNA (ssDNA) molecules with identical nucleotide sequences (“4”) could be acquired, marking the initial stage of amplification. One component of “4” served as the trigger sequences, which facilitated the ongoing production of additional quantities of ssDNA by EXPAR. Interestingly, the others could be utilized to partake in the subsequent CHA. The CHA was then triggered by the addition of the designed L1 and L2. The L2 probe initially obstructed the G-rich segment while the L1 and L2 were in a closure state. The CHA method yielded an L1/L2 duplex with the G-rich regions exposed. The G-rich segment has the ability to form a G-quadruplex structure, which interacts with ThT and ultimately enhances the fluorescence signal.

## Materials and Methods

### Chemical and Reagents

Nt.BbvCl nicking enzyme, DEPC-treated deionized water, Klenow Fragment Polymerase, Deoxyribonucleotide triphosphates (dNTPs) were purchased from New England BioLabs (China). Streptavidin (SA) and MNPs were purchased from Sigma-Aldrich (USA). Thioflavin T (ThT) was purchased from Shanghai Aladdin Biochemical Technology Co., Ltd. (China). Phosphate buffer solution was purchased from Beijing Solibao Technology Co., Ltd. (China). SYBR Green I nucleic acid dye was purchased by Tiangen Biochemical Technology Co., Ltd. (China). The sequences of DNA used in EXPAR and CHA were shown as Table S1: All sequences were synthesized by Sangon Bio. Tech. (China).

### Surface Modification of Magnetic Nanoparticles with F23 Aptamers

The magnetic nanoparticles were cleansed with PBS (0.01 M, pH 7.4) prior to their application in the subsequent procedures: The beads were suspended in the centrifuge tubes through the gentle vortexing. After positioning the tubes on the magnetic separation rack, the tubes contained liquid, which was subsequently extracted using a pipette gun. In order to re-suspend the beads, remove the tubes from the magnetic separation rack, add fresh PBS, and gently vortex or rotate the centrifuge tube.

The F23 aptamers were diluted in PBS to a concentration of 10 nM. Next, 100 μl of streptavidinized magnetic nanoparticles (MNPs) with a concentration of 5 mg/ml were combined with 100 μl of biotinylated estradiol aptamers with a concentration of 10 nM. The mixture was then allowed to react at a temperature of 25°C for a duration of 1.5 h. In this instance, the magnetic nanoparticles and the aptamers were connected through the streptavidin-biotin interaction to create a complex known as MNPs-Apt. Following the process, the mixtures were extracted by magnetic separation. The nanoparticles underwent three rounds of washing with PBS buffer solution, were then re-suspended in PBS, and stored at a temperature of 4°C for future use.

### Detection Procedures

(1) Recognition: The modified capture@MNPs (100 μl) mentioned above were combined with a range of target standards that varied in concentration (10 μl). The mixture was incubated at 37°C for 60 min. The liquid portion was collected using magnetic separation prior to the 60-min reaction at 37°C.

(2) EXPAR: The 51 μl of supernatant from (1) was utilized as primers. Subsequently, template DNA (0.2 ml, 2 μM) and incision endonuclease buffer (5 μl) were added to the supernatant and incubated at 90°C for 10 min. Once the mixture has cooled down gradually to the temperature of the surrounding environment, 1 μl of Nt.BbvCI nicking enzyme (0.8 U/μl), 1 μl of Klenow Fragment DNA polymerase (0.05 U/μl), Klenow Fragment buffer (5 μl), and dNTPs (0.5 μl, 10 μM) were added. The volume was restored to 50 μl using DEPC treated deionized water. Subsequently, the mixes were placed in an incubator set at a temperature of 37°C for a duration of 50 min. The enzyme was deactivated by maintaining the combinations at a temperature of 80°C for a duration of 20 min. (3) CHA signal output: 5 μl ThT, L1 (50 μl, 1 μM) and L2 (50 μl, 1 μM) were added to the solution in (2) and oscillated at 37°C for 1 h. The reaction products were measured by fluorescence spectrophotometer.

## Results and Discussion

### The Working Mechanism of the Proposed Method for Sensitive *P. aeruginosa* Detection

**Fig. 2:**
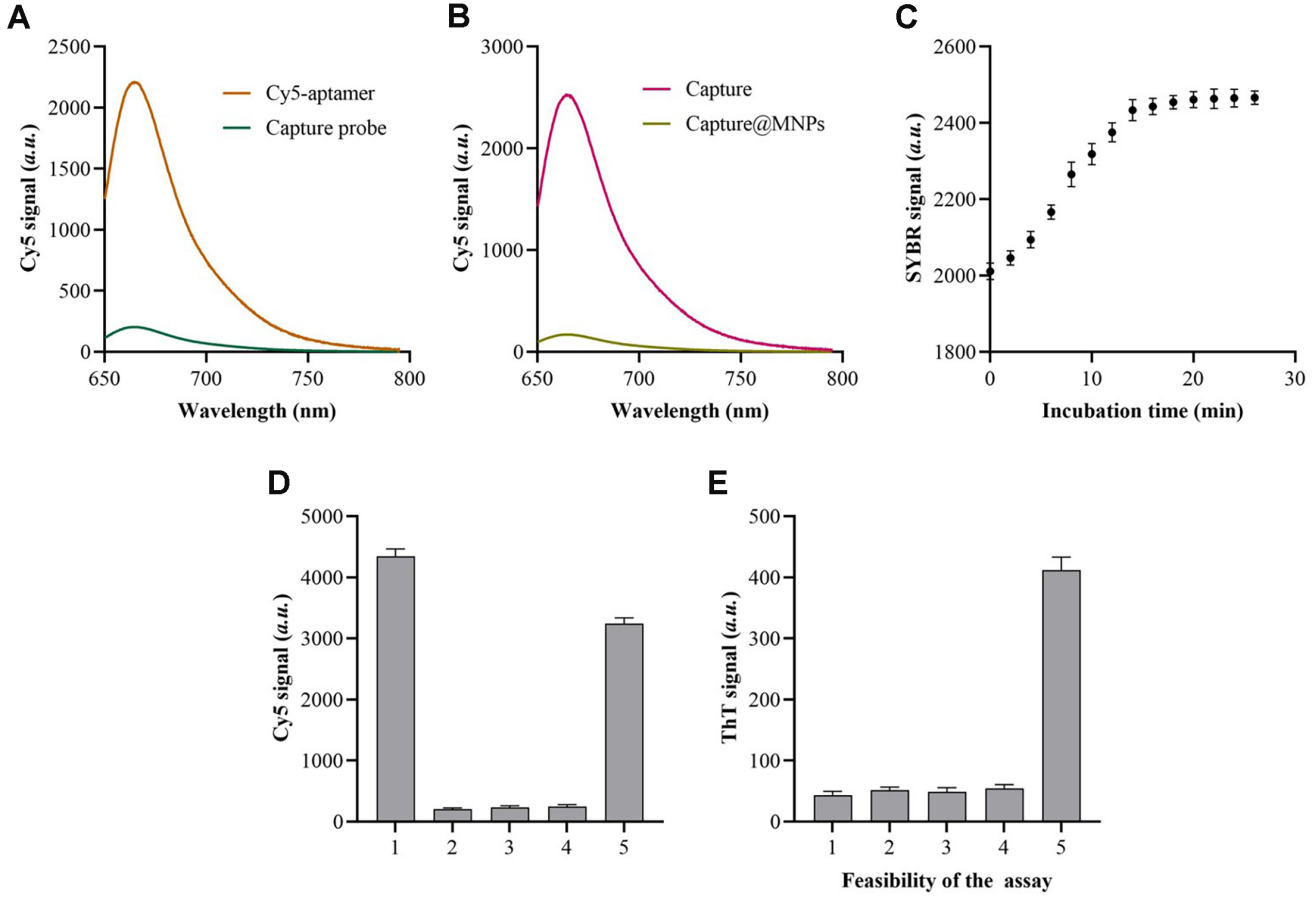
Feasibility of the proposed method for *P. aeruginosa* detection. (**A**) Fluorescence spectrum of Cy5 labeled aptamer before and after being assembled into capture probe. (**B**) Fluorescence spectrum of Cy5 labeled capture probe before and after being fixed on the surface of MNPs. (**C**) SYBR Green I signals of the EXPAR process with different incubation time. (D) Cy5 signals of the L2 probe during the CHA process. column 1: L2 probe in linear state, column 2: L2 probe, column 3: L2+ L1, column 4: L2+ “4”, column 5: L2+ L1+ “4”. (E) ThT signals of the method when essential components existed or not. column 1: target (-), column 2: p-EXPAR (-), column 3: CHA (-), column 4: ThT (-), column 5: with all. Data were expressed as mean ± standard deviations, *n* = 3 technical replicates.

The capture probe is constructed using the F23 aptamer and “2”. In order to confirm the construction of the capture probe, the 5' ends of the F23 aptamer and the 3' ends of the “2” sequence were tagged with Cy5 and BHQ, respectively. As depicted in Fig. 2A, the notable fluorescence signal of Cy5 was much diminished following the assembly of the capture probe. Furthermore, the assembly of capture@MNPs is confirmed by attaching Cy5 labels to the 5' ends of the aptamer in the capture probe. As depicted in Fig. 2B, in the absence of MNPs, the fluorescence intensity of the supernatant is significantly elevated. Upon the assembly of capture@MNPs, the Cy5-capture probe component was isolated, resulting in a notable decrease in fluorescence intensity in the supernatant.

The EXPAR method was subsequently verified using real-time fluorescence monitoring. Fig. 2C clearly demonstrates a gradual increase in the fluorescence of SYBR Green I over time. The SYBR Green I signals reached saturation and stopped increasing after the EXPAR was conducted for more than 20 minutes. This indicates that all TS sequences were occupied by the created “4” chains.

Fluorescence experiments were employed to validate the CHA method. As depicted in Fig. 2D. the presence of L1 and L2 leads to a decrease in the fluorescence intensity of Cy5, suggesting that the L2 probe retains the stem-ring structure under these conditions. Significant fluorescence intensity is found when the synthetic “4” sequence is present, indicating the activation of CHA (*P* < 0.05). This activation leads to the opening of a large number of L2 probes, which in turn releases the G-rich area and triggers the signal response. The specificity of the p-EXPAR and CHA process was further evaluated. As shown in Fig. S1, the p-EXPAR method showed a higher selectivity to the sequences with one (M1) or two (M2) mismatched bases. In addition, the Fig. S2 indicated the high specificity of the CHA process.

The validity of this approach was confirmed through fluorescence experiments. The fluorescence was at its lowest level when both the target (column 1) and EXPAR (column 2) were absent, as seen in Fig. 2E. However, the fluorescence intensity exhibited a substantial rise when target, EXPAR, and CHA were present simultaneously (column 5, *P* < 0.05). Without the CHA process (column 3) and ThT (column 4), the fluorescence exhibited a small rise. This could be attributed to the non-specific hybridization occurring between interfering sequences and the template in EXPAR.

### Optimization of Detection Parameters

**Fig. 3:**
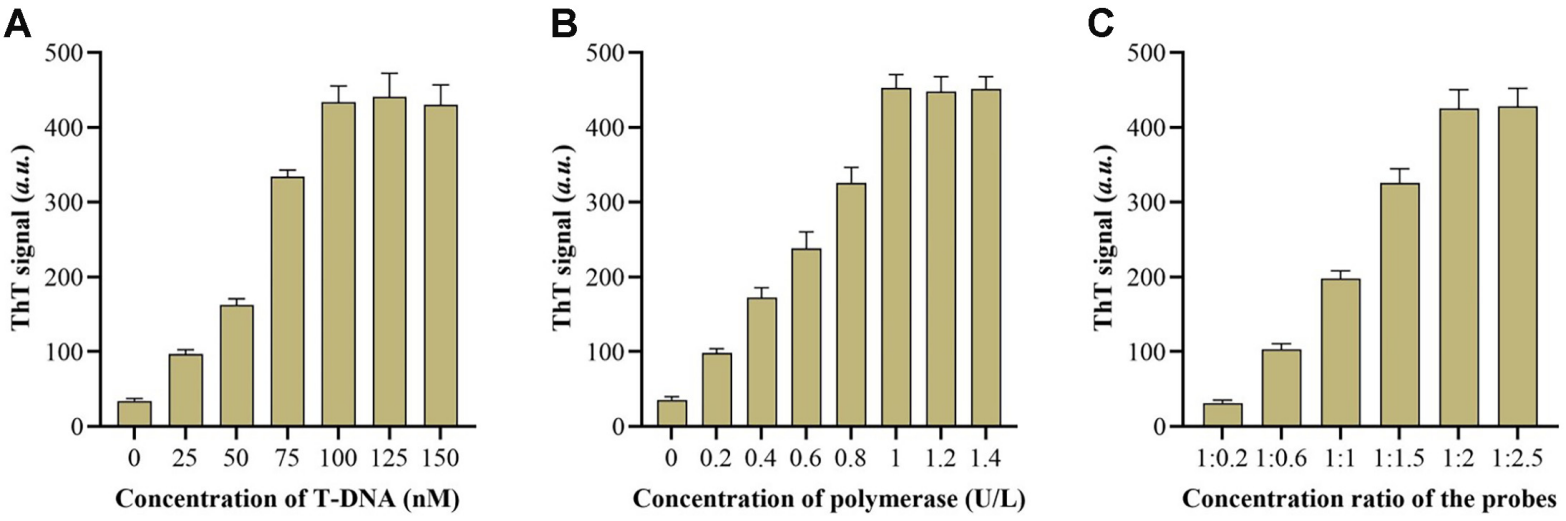
Optimization of experimental parameters. ThT signals of the method when detecting under different T-DNA concentrations (**A**) polymerase concentrations (**B**) and concentration ratio of the L probes (**C**). Data were expressed as mean ± standard deviations, *n* = 3 technical replicates.

The optimal fluorescence response was achieved by evaluating multiple factors in the reaction process, including T-DNA, Klenow Fragment polymerase, and the ratio of L1 to L2. Under ideal conditions, it is expected that the detection method would exhibit increased sensitivity and stability. Fig. 3A illustrates the optimization of the quantity of T-DNA for EXPAR. Fluorescence intensity was significantly low when the quantity of T-DNA was either excessive or insufficient (*P* < 0.05). The insufficient amount of T-DNA resulted in inadequate amplification, whereas an excessive amount of T-DNA caused the merging of individual strands and templates, hence impeding the induction of CHA. Hence, a concentration of 100 nM was determined to be the most suitable quantity of T-DNA to be added. The optimal amount of polymerase added by the Klenow Fragment is presented in Fig. 3B. Insufficient polymerases in the EXPAR amplification resulted in a fluorescence intensity below the typical level. Nevertheless, an abundance of polymerase led to a reduction in fluorescence recovery (*P* < 0.05). The inhibition of nicking enzymes and the disruption of EXPAR amplification may have been caused by an overabundance of polymerases. After considering cost savings and reaction efficiency, we determined that adding 1 U/L of Klenow Fragment polymerase is the most optimal choice.

From Fig. 3C, it is evident that when the proportion of L1 and L2 grew, there was a gradual increase in the recovery of fluorescence intensity. The alteration became apparent only after adding 1:2. In the end, the ideal additions for L1 and L2 were decided as 1:2 (*P* < 0.05). Increasing the concentration of L2 probe is crucial for both the CHA and the signaling reply.

### Application of the Method for *P. aeruginosa* Detection

**Fig. 4:**
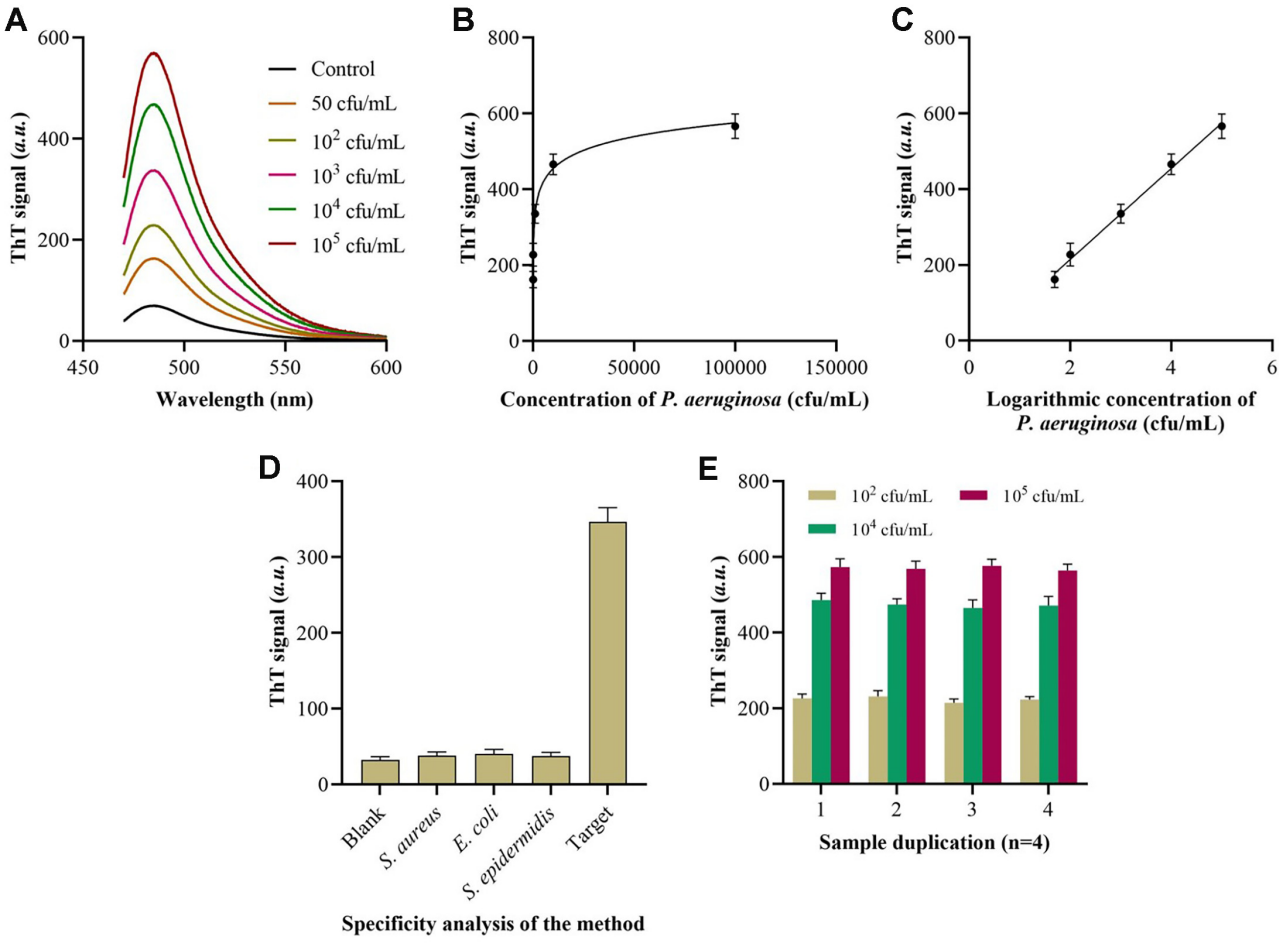
Analytical performance of the proposed method for *P. aeruginosa* detection. (**A**) Fluorescence spectrum of the method for different concentrations of target bacteria. (**B**) Correlation between the ThT signals and the concentrations of bacteria. (**C**) Linear correlation equation between the ThT signals and the logarithmic concentrations of bacteria. (**D**) ThT signals of the method for different bacteria detection. (**E**) ThT signals of the method when detecting sample duplicates.

As depicted in Fig. 4A, the fluorescence intensity value exhibited a progressive increment in correlation with the increase in the target concentration. Hence, we can accurately measure the concentration of the desired substance by analyzing the fluorescence intensity values at a wavelength of 490 nm. Based on the data presented in Fig. 4B and 4C, there is a strong linear correlation observed within the concentration range of 50 CFU/ml to 10^5^ CFU/ml. The limit of detection is 16 CFU/ml (S/N=3), which is comparable to or better than the methods previously described.

Subsequently, we examined the degree of specificity exhibited by the biosensor. Fig. 4D clearly demonstrates a noticeable disparity in the signal when *P. aeruginosa* is present, whereas the signal from the four negative control bacterial strains was consistently below 20% of the signal from *P. aeruginosa*. Hence, the biosensor exhibits exceptional specificity in identifying *P. aeruginosa*. Ultimately, the assessable quality of the biosensor was determined. As depicted in Fig. 4E, the fluorescence signals were observed on five occasions using *P. aeruginosa* concentrations of 10^2^, 10^4^, and 10^5^ CFU/ml. The standard deviations for each variable were 3.43%, 3.17%, and 3.62%, respectively. The results demonstrated that the biosensor exhibited exceptional reproducibility and stability.

### *P. aeruginosa* Analysis in Clinical Samples

In order to assess the possible clinical applicability of the biosensor, recovery experiments were conducted using commercially available serum samples. The serum samples were added with specific amounts of *P. aeruginosa*. The urine samples were subsequently identified using the developed biosensor. The test settings for the samples were identical to those used for the standard samples, and each measurement was replicated five times. The recovery rates, as indicated in Table 1, were computed to be between 97.12% and 104.2%, with relative standard deviations varying from 2.65% to 4.12%. These findings suggest that the biosensor has significant promise for clinical applications in complex biological samples.

## Conclusion

To summarize, we have created a new method for detecting *P. aeruginosa* using a fluorescence amplification strategy that relies on aptamers. This method achieves great sensitivity by amplifying the signals with p-EXPAR and CHA. Furthermore, our two-step isothermal amplification approach demonstrated excellent analytical performance, remarkable sensitivity, selectivity, and stability in detecting *P. aeruginosa*. In comparison to previous research, the method demonstrated a limit of detection as low as 16 CFU/ml and a broader detection range. This suggests that the technology is capable of accurately analyzing trace amounts in complicated samples. In addition, the p-EXPAR method was demonstrated to show a higher selectivity to mismatched sequences compared with the EPXAR, indicating that the p-EXPAR possesses a higher selectivity. This method proved to be effective, sensitive, and convenient due to its relatively simple operating stages, regulated reaction conditions, and ability to detect genuine samples with high sensitivity. As anticipated, this pragmatic approach was also projected to be utilized in the examination and precise measurement of proteins and nucleic acids, offering a novel concept for the on-site detection of impurities in healthcare. In the future, we will integrate the proposed sensing system into portable devices, such as microfluidics, for point-of-care testing.

## Supplemental Materials

Supplementary data for this paper are available on-line only at http://jmb.or.kr.



## Figures and Tables

**Table 1:** Recovery rate of the method for detection from constructed clinical samples (*n* = 5).

Title	Original amount	Detected amount	Rate	RSD
1	100	104.2	104.2%	3.54%
2	1000	971.2	97.12%	4.12%
3	5000	5154	103.1%	2.65%

RSD, relative standard deviations.
