# Supplementary material for: A Label-Free Fluorescent Amplification Strategy for High-Sensitive Detection of Pseudomonas aeruginosa based on Protective-EXPAR (p-EXPAR) and Catalytic Hairpin Assembly
Source: J Microbiol Biotechnol. 2024 Jun 14;34(7):1544–9. doi: 10.4014/jmb.2405.05006 (PMC11294642; doi:10.4014/jmb.2405.05006)
Supplement: Supplementary file 1 [file jmb-34-7-1544-supple.pdf]

J. Microbiol. Biotechnol. 2024. 34(7): 1544–1549
https://doi.org/10.4014/jmb.2405.05006

Supplementary Table and Figures

A Label-Free Fluorescent Amplification Strategy for High-Sensitive Detection of *Pseudomonas aeruginosa* based on Protective-EXPAR (p-EXPAR) and Catalytic Hairpin Assembly

Table S1. Oligonucleotide sequences.

Title	Sequences (5' to 3')
T-DNA	CTT CCT GCT TAT TGA ATT ACG CTG AGG GCT AGA GAT TTT CCA CAC TGA CTT CCT GCT TAT TGA ATT ACG CTG-P
2	CAG CGT AAT TCA ATA AGC AGG AAG
L1	GCT AGA GAT TTT CCA CAC TGA CTT GTT GGG TTT TGG GAG TCA GTG TGG AAA
L2	ACT GAC TCC CAA AAC CCA ACA AGT CAG TGT GGA AAA TCT TGT TGG GTT TTG GGT TTT GGG TTT TGG G

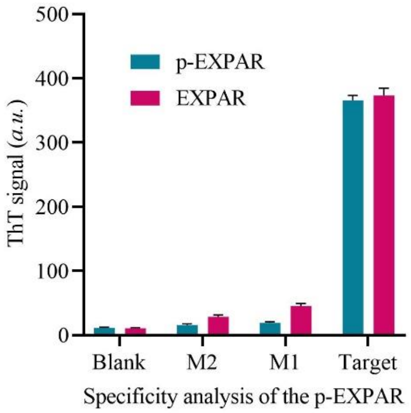

Fig S1. The ThT signals of the p-EXPAR and EXPAR to the mismatched sequences detection.

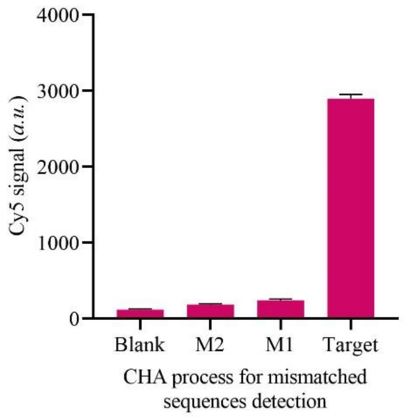

Fig S2. The Cy5 signals of the L2 probe in CHA process when detecting the mismatched sequences.
